# Interplay Between DNA Polymerase, RNA Polymerase, and RNase H1 During Head-On Transcription–Replication Conflict

**DOI:** 10.3390/ijms262311515

**Published:** 2025-11-27

**Authors:** Nadezhda A. Timofeyeva, Ekaterina I. Tsoi, Darya S. Novopashina, Nikita A. Kuznetsov, Aleksandra A. Kuznetsova

**Affiliations:** 1 Institute of Chemical Biology and Fundamental Medicine, Siberian Branch of Russian Academy of Sciences, 630090 Novosibirsk, Russia; e.tsoi1@g.nsu.ru (E.I.T.); danov@1bio.ru (D.S.N.); nikita.kuznetsov@1bio.ru (N.A.K.); 2 Department of Natural Sciences, Novosibirsk State University, 630090 Novosibirsk, Russia

**Keywords:** transcription-replication conflict, RNA polymerase, DNA polymerase, RNase H1, R-loop, enzymatic activity, enzyme kinetics

## Abstract

Transcription–replication conflicts (TRCs) often occur in cells and cause DNA replication fork stalling. In this study, we investigated the interplay of RNA polymerase (RNAP), DNA polymerase, and RNase H1 (RH1) during head-on TRC in vitro with precise control over the reaction conditions. We show that it is a catalytically competent transcription elongation complex (TEC) that interferes with the action of both the Klenow fragment and full-length DNA Pol I. An incompetent RNAP complex with an R-loop stimulates the 3′→5′ exonuclease activity and pauses the DNA polymerase during head-on TRC. As RNAP advances along the DNA template, elongating the RNA, the head-on TRC is slowly overcome in our model system, likely through the reassociation of the displaced DNA polymerase with the nontemplate DNA strand upstream of RNAP. An isolated R-loop containing an 11-nt heteroduplex (R-loop-11) does not interfere with DNA replication by the Klenow fragment. For DNA Pol I, such an R-loop also does not stall replication but stimulates its 3′→5′ exonuclease activity. We demonstrate that a stalled Klenow fragment does not interfere with transcription, whereas a Klenow fragment moving along the TRC substrate towards RNAP alters the kinetics of RNAP. Stalled DNA Pol I does not stop RNAP but stimulates its endonuclease activity. We find that RH1 alone does not displace stalled RNAP from a competent TEC containing R-loop-11 and does not resolve the head-on TRC. On the other hand, RH1 displaces RNAP from the incompetent complex with the TRC substrate. This eliminates the stimulation of the 3′→5′ exonuclease activity of DNA polymerase during head-on TRC.

## 1. Introduction

The processes of replication and transcription can occur simultaneously on the same DNA molecule, leading to transcription–replication conflicts (TRCs). TRCs can be either co-directional or head-on. A conflict is called co-directional if the DNA polymerase moves in the same direction as the RNA polymerase, and head-on if the two polymerases move towards each other. Both head-on and co-directional conflicts can cause stalling of the DNA replication fork [1,2,3,4], which can lead to DNA damage and genomic instability. Therefore, the ability to avoid and resolve TRCs is essential for the survival of both prokaryotic and eukaryotic cells [5,6].

In prokaryotic cells, replication and transcription occur simultaneously, making the temporal separation of these processes impossible. In eukaryotic cells, however, replication is confined to the S phase, while transcription occurs throughout all phases of the cell cycle [7,8]. Therefore, one might expect that this partial temporal separation would reduce the frequency of TRCs. However, this separation does not result in a dramatic reduction in TRCs, probably due to the persistence of RNA polymerases on DNA throughout replication [9].

Head-on TRCs have been shown to impede replication fork progression more than co-directional TRCs [10]. This is likely the reason why the genomes of many species are evolutionarily organized to avoid head-on conflicts in the case of important and highly transcribed genes. In bacteria, such genes are mainly located on the leading DNA strand; therefore, their transcription occurs co-directionally with replication [5]. In *S. cerevisiae*, the eukaryotic model system for TRC studies, the tRNA genes are also predominantly located on the leading strand, although a significant proportion of *S. cerevisiae* genes are still located on the lagging strand [11]. However, in human cells, replication initiation events are largely stochastic [12]. Thus, neither eukaryotic nor prokaryotic cells can completely avoid head-on TRCs. Consequently, many auxiliary factors are involved in resolving TRCs to sustain genomic stability [3,4,13].

Replication plays a more important role in cell survival than transcription. Consequently, the degradation of the transcription elongation complex (TEC) promotes the prevention and resolving of TRC without seriously compromising genomic integrity. The TEC is a complex of RNA polymerase (RNAP) and an R-loop structure, which consists of a DNA–RNA heteroduplex and a displaced strand of DNA [14]. Typically, the length of the DNA–RNA heteroduplex within the TEC does not exceed 10 bp in length, a feature controlled by a special wedge-like structure of RNAP [15,16]. RNAP can form several types of complexes with an R-loop. In a catalytically competent TEC, the 3′ terminal nucleotide of the nascent RNA and the unpaired nucleotide of the template DNA are positioned in the RNAP active site. This state of the enzyme–substrate complex is known as the post-translocated state. The incorporation of a nucleotide into the nascent transcript shifts the complex to the pre-translocated state. The nucleic acid scaffold then translocates relative to the RNAP main frame, reforming the post-translocated state. This oscillation of TEC between the pre-translocated and post-translocated states, coupled with proper nucleotide incorporation, drives the forward movement of RNAP [14]. In addition, RNAP can move backward (in the direction opposite to transcription). This backward movement extrudes the 3′ terminal nucleotide of the nascent RNA from the active site, forming states backtracked by one or more nucleotides. A transition to a state backtracked by two nucleotides allows for the endonucleolytic cleavage of the transcript by RNAP, which restores the catalytically competent TEC. Alternatively, RNAP can move backward without RNA cleavage, forming stable, incompetent backtracked complexes with R-loops [17]. Numerous factors destabilize the TEC to clear the DNA for the replisome. For example, the transcription repair coupling factor Mfd displaces RNA polymerase stalled on damaged DNA and recruits DNA excision repair factors. A similar function has been proposed for the transcription factor CarD from *Mycobacterium tuberculosis*. The small signaling nucleotide guanosine tetraphosphate (ppGpp), which is produced under stress conditions in bacteria, displaces stalled RNA polymerase from complexes with damaged DNA. Furthermore, co-directional conflicts can be resolved without degradation of the TEC. For instance, the transcript cleavage factors GreA and GreB can restore the activity of a stalled, backtracked TEC by stimulating the cleavage of the displaced 3′-end of the RNA transcript [5,10,17,18].

As transcription proceeds, the transient DNA–RNA heteroduplex unpairs as the nascent RNA is released through the RNA exit channel. However, stable R-loops can form behind RNAP if the nascent RNA re-hybridizes with the upstream template DNA strand. This process is promoted by negative supercoiling accumulating directly behind the moving RNAP [19]. R-loop formation is considered a major cause of transcription–replication conflicts (TRCs). Furthermore, head-on collisions between the transcription and replication machineries increase R-loop levels, whereas co-directional collisions decrease them [20]. Transcription-coupled R-loops stall replication forks and cause genome rearrangements in both prokaryotic and eukaryotic cells [21,22]. R-loops are primarily removed by RNase H1 (RH1), an enzyme that degrades the RNA strand within the RNA-DNA heteroduplex, making RH1 one of the important participants in preventing or resolving TRCs [5,13].

The present study was devoted to an analysis of the interplay between recombinant RNAP, DNA polymerase (both the Klenow fragment and full-length DNA Pol I), and RH1 from *E. coli*. We designed a model TRC substrate that mimics the nucleic acid structure arising during a head-on transcription–replication conflict, containing both an R-loop and a replication fork. We analyzed the efficacy of the formation of competent enzyme–substrate complexes between the designed substrate and RNAP or DNA polymerases and determined the rates of RNA or DNA synthesis. Furthermore, we assessed the ability of RH1 to resolve the model TRC in the presence of recombinant RNAP and DNA polymerases. It should be noted that the in vivo replisome is far more complex than the model system used here. Many additional proteins are associated with the replisome, including DnaB helicase and Rep helicase, which likely facilitate the removal of barriers in front of the fork. Furthermore, the main replicative DNA polymerase in *E. coli* is Pol III. In addition, the above-mentioned factors Mfd, GreA, and GreB facilitate the removal of stalled RNAPs. The inclusion of all these proteins was beyond the scope of this study and their examination in our model system is the subject of further research.

## 2. Results and Discussion

### 2.1. The Rationale for Model TRC-Substrates

**Figure 1:**
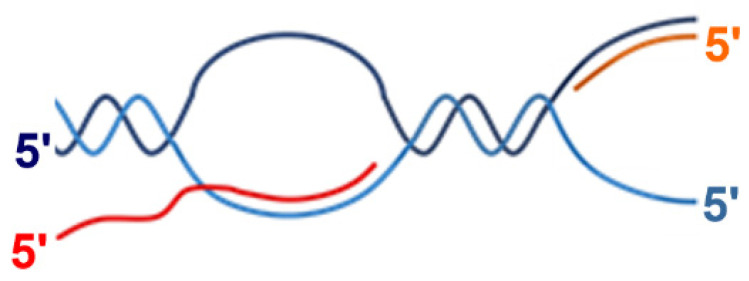
Schematic presentation of the substrate containing an R-loop and a replication fork used in this study (TRC-substrate-11). The template DNA strand is indicated by light blue, the nontemplate DNA strand is indicated by dark blue, the **RNA polymerase** (RNAP) primer is indicated by red, the DNA polymerase primer is indicated by orange.

In this study, we designed a substrate that mimics the nucleic acid structure arising during a head-on TRC (TRC-substrate-11, Table 1, Figure 1). This substrate contains an R-loop and a replication fork (RF). The length of the RNA–DNA heteroduplex (and the bubble size) in the R-loop was 11 nt. The 11-nt heteroduplex length slightly exceeds the size of natural heteroduplexes inside the TEC (typically 8–10 nt). A 25-nt deoxyribooligonucleotide was used as a primer for DNA polymerase. To establish a basis for comparison of RNAP action in the presence and absence of DNA polymerase and the DNA primer, we used a substrate containing the R-loop but lacking the primer for DNA polymerase (R-loop-substrate-11). Furthermore, to establish a basis for comparison of DNA polymerase action in the presence and absence of RNAP, the R-loop, or the template DNA strand, we used a substrate containing the replication fork but lacking the RNA primer (RF-substrate-11), as well as a substrate without the RNA primer and the template DNA strand (Control-substrate-11). Substrates containing a FAM-labeled RNA primer were used to visualize the actions of RNAP or RH1. Substrates containing a FAM-labeled DNA primer were used to visualize DNA polymerase action.

### 2.2. RNA Extension by RNAP in the TRC Substrate

**Figure 2:**
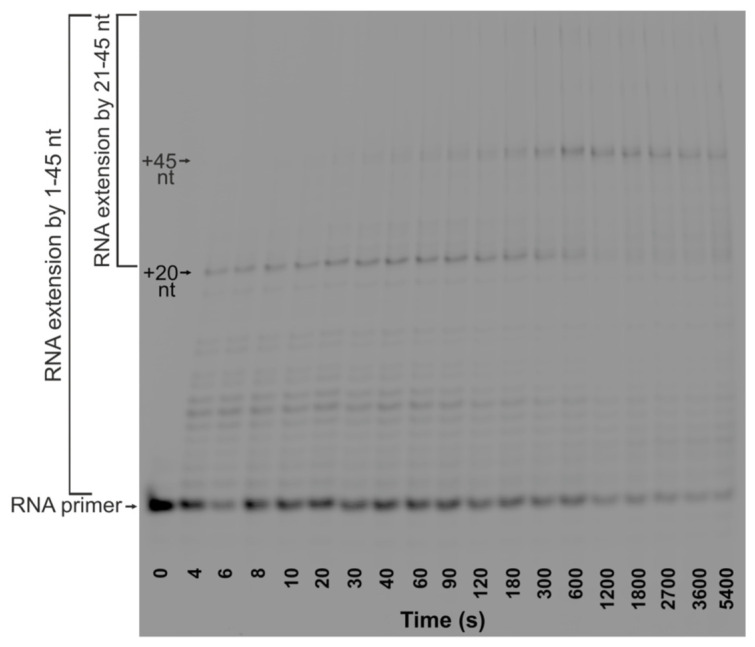
Representative polyacrylamide gel electrophoresis (PAGE) analysis of RNA products. The time course of RNA extension in TRC-substrate-11 (0.5 μM) by RNAP (1 μM) in the presence of Klenow fragment (1 μM), NTPs (100 μM), and dNTPs (100 μM).

**Figure 3:**
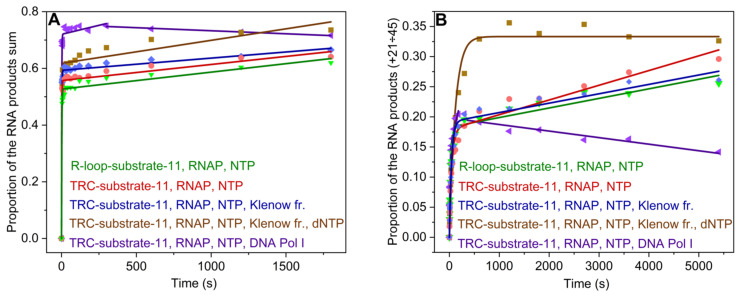
The time courses of RNA extension by RNAP by 1–45 nt (**A**) and 21–45 nt (**B**) in R-loop-substrate-11 (green), in TRC-substrate-11 (red), in TRC-substrate-11 bound to Klenow fragment without dNTPs (blue), in TRC-substrate-11 in the presence of Klenow fragment and dNTPs (brown), in TRC-substrate-11 bound to DNA Pol I without dNTPs (purple). Concentration of the enzymes was 1 μM, and that of TRC-substrate-11 was 0.5 μM. Smooth curves are the results of the fitting procedure.

To determine the efficiency of TEC formation and the rate of RNA extension by RNAP in the TRC complexes, kinetic traces of the accumulation of total RNA products from +1 to +45 nt in the presence of Klenow fragment or DNA Pol I were obtained (Figure 2 and Figure 3A). The reaction mixtures either contained the dNTPs or did not. In addition, to establish the basis for comparison of RNAP action in the presence and absence of DNA polymerase, we obtained kinetic traces of accumulation of RNA products in the absence of DNA polymerase and DNA primer (in R-loop-substrate-11 which contains the R-loop but lacks the DNA polymerase primer) or in the absence of DNA polymerase alone (in TRC-substrate-11) (see Table 1).

We have determined the dissociation constant characterizing the stability of the entire RNAP complex with the R-loop containing the 9 nt bubble (R-loop-9) to be 0.3 μM using a microscale thermophoresis assay (see ref. [23]). This dissociation constant characterizes the entire RNAP complex with R-loop-9 irrespective of whether it is catalytically competent or not. Furthermore, we have determined the dissociation constant characterizing the stability of only the catalytically competent RNAP complex with R-loop-9 to be 0.8 μM from the dependence of the catalytically competent TEC proportion on RNAP concentration (see Supplementary Materials). The dissociation constant obtained for the catalytically competent TEC was 2.7-fold higher than the dissociation constant of the overall RNAP complex with the R-loop. Based on these data, we have concluded that the concentration of the catalytically competent TEC is lower than that of the entire RNAP complex with the R-loop at the initial moment. We have proposed that some proportion of the RNAP complexes with an R-loop are initially in an active conformation, forming the catalytically competent TEC, while the remainder are initially in a catalytically inactive conformation (probably a backtracked conformation). Through mutual conformational changes (induced fit), the inactive complex can transform into a catalytically competent TEC (see ref. [23]).

Given the defined dissociation constants, in the present study, we used micromolar concentrations of RNAP to obtain detectable binding of RNAP to the substrate.

The time courses of the accumulation of total RNA products in the absence of DNA polymerase or in the presence of Klenow fragments were fitted to Equation (2): burst exponential growth was followed by linear growth (see Section 3.4, Figure 3A). It was found that the presence of a DNA primer alone, or a DNA primer together with the Klenow fragment, does not affect significantly the initial concentration of the catalytically competent TEC ([TEC]^Sum^) (corresponding to TEC formation efficiency), the rate of the burst phase ($k_{1}^{RNA\;Sum}$) (corresponding to RNA synthesis in a processive mode within the catalytically competent TEC), or the rate of the slow linear phase ($k_{2}^{RNA\;Sum}$) (corresponding to induced fit within the inactive complex of RNAP with an R-loop, leading to the formation of catalytically competent TEC, as was suggested in [23]), since these parameters were very similar among all kinetic traces obtained in the absence of dNTPs (Table 2). However, the addition of dNTPs to the reaction mixture with the Klenow fragment increases the rate of induced fit within the inactive complex of RNAP with an R-loop ($k_{2}^{RNA\;Sum}$) almost two-fold. This result is presumably associated with the DNA primer extension by DNA polymerase leading to the separation of DNA strands in TRC-substrate-11 during the movement of RNAP and DNA polymerase towards one another. The kinetic trace of the accumulation of total RNA products in the presence of DNA Pol I also showed an increase in the product fraction within the time range from 0 to 300 s (Figure 3A). Moreover, a kink in the kinetic trace was observed at ~300 s, followed by a slow linear decrease in the total product fraction. Therefore, in the presence of DNA Pol I, the time course was fitted to Equation (2) (single-exponential growth followed by linear growth) within the time range from 0 to 300 s. The further linear decrease was fitted to linear Equation (5). It could be suggested that head-on collision of RNAP with full-length DNA Pol I stimulates endonuclease degradation of RNA, which results in a decrease in the total amount of RNA products. This RNA degradation is characterized by the observed rate constant $k_{endo}^{RNA\;Sum}$ (Table 2).

Since the replication fork within the TRC-substrate-11 is located at a distance of 20 nt from the 3′ end of the RNA primer, we analyzed the accumulation of RNA products from +21 to +45 nt (Figure 3B). The time courses of accumulation of RNA products from +21 to +45 nt were fitted to Equation (2) (single-exponential growth followed by linear growth) in the presence of the Klenow fragment or to single-exponential Equation (1) in the presence of the Klenow fragment together with dNTPs. The amplitude of the burst phase ([TEC]^21÷45^) represented the initial proportion of TEC, where the RNA primer was elongated in the region of the replication fork. As can be seen from Table 2, the presence of the Klenow fragment barely altered this amplitude as well as the observed burst rate constant $k_{1}^{RNA\;[21÷45]}$ (characterizing the pre-steady-state accumulation of long RNA products) and the observed rate constant of the slow phase $k_{2}^{RNA\;[21÷45]}$ (characterizing the steady-state accumulation of long RNA products). These data indicate that Klenow fragment, stalled in the complex with the replication fork, does not stop transcription in the model system of head-on transcription–replication conflict.

In the presence of the Klenow fragment together with dNTPs, the time course of the accumulation of RNA products from +21 to +45 nt was fitted to single-exponential Equation (1). From the time courses presented in Figure 3B, it is evident that RNA products from +21 to +45 nt accumulate at a similar rate during the first 120 s in the presence and absence of dNTPs. Consequently, the rate constants of the pre-steady-state accumulation of long RNA products ($k_{1}^{RNA\;[21÷45]}$) in the presence and absence of dNTPs should be similar. At the same time, in the presence of dNTPs, the time course was fitted to a single-exponential equation and yielded the observed rate constant that was 2.7-fold lower than $k_{1}^{RNA\;[21÷45]}$ in the absence of dNTPs. Therefore, the observed rate constant of the accumulation of RNA products from +21 to +45 nt in the presence of the Klenow fragment, together with dNTPs, characterizes the steady-state accumulation of long RNA products $k_{2}^{RNA\;[21÷45]}$ (Table 2). The rate constant $k_{1}^{RNA\;[21÷45]}$ cannot be determined in the latter case, since $k_{1}^{RNA\;[21÷45]}$ and $k_{2}^{RNA\;[21÷45]}$ are of the same order of magnitude. Thus, the addition of dNTPs to the reaction mixture with the Klenow fragment increased [TEC]^21÷45^ nearly two-fold and $k_{2}^{RNA\;[21÷45]}$ by more than two orders of magnitude. It can be concluded that the movement of DNA polymerase towards RNAP (in the presence of dNTPs) increases the rate of steady-state accumulation of long RNA products and the TEC proportion where the RNA primer is elongated up to the edge of the DNA template, probably due to a decrease in paused TECs proportion caused by the separation of DNA strands during the movement of RNAP and DNA polymerase towards one another.

The kinetic trace of the accumulation of RNA products in TRC-substrate-11 from +21 to +45 nt in the presence of full-length DNA Pol I showed an increase in the product fraction within the time range from 0 to 180 s with further kinks, followed by a slow linear decrease in RNA product fraction (Figure 3B). The time course was fitted to Equation (2) (single-exponential growth followed by linear growth) within the time range from 0 to 180 s and yielded the observed rate constants $k_{1}^{RNA\;[21÷45]}$ and $k_{2}^{RNA\;[21÷45]}$. The further linear decrease was fitted to linear Equation (5) from 300 s and yielded the observed rate constant $k_{endo}^{RNA\;[21÷45]}$, characterizing the endonuclease degradation of long RNA products (Table 2). Consequently, in the presence of DNA Pol I, the equilibrium accumulation of RNA products from +21 to +45 nt shifted backwards, as did the equilibrium accumulation of total RNA products. Thus, stalled DNA Pol I seems to stimulate the endonuclease activity of RNAP in the complex with TRC-substrate-11. These results correlate with data described in [17], demonstrating that the probability of RNAP backtracking increases if there is an obstacle such as the DNA-bound protein. On the other hand, our data revealed that an obstacle such as DNA Pol I (or its Klenow fragment) is not itself a permanent block to transcription, since RNAP transcripts reach +45 nt in the presence of stalled DNA polymerase I. During head-on TRC in vivo, DNA between the TEC and the replication fork is supercoiled. This supercoiling can cause RNAP backtracking and stalling. In the model system used in this study, the supercoiling is absent. Thus, when RNA-elongating TEC collides with DNA polymerase, it remains competent and can displace the DNA polymerase.

Thus, the obtained results reveal that the transcription is not interfered with by the Klenow fragment, which is stalled in the complex with the replication fork in our model system of head-on TRC. The Klenow fragment, which moves along the TRC substrate containing the 11-nt bubble (TRC-substrate-11) towards RNAP, elongating the DNA, accelerates the induced fit within the inactive complex of RNAP with an R-loop leading to the formation of the catalytically competent TEC. In addition, it increases the TEC proportion where the RNA primer is elongated up to the edge of the DNA template and the rate of steady-state accumulation of long RNA products, probably due to a decrease in paused TECs proportion. On the other hand, head-on collision of RNAP with full-length DNA Pol I does not stop RNAP but stimulates its endonuclease activity.

### 2.3. DNA Extension by DNA Polymerase in the TRC-Substrate

**Figure 4:**
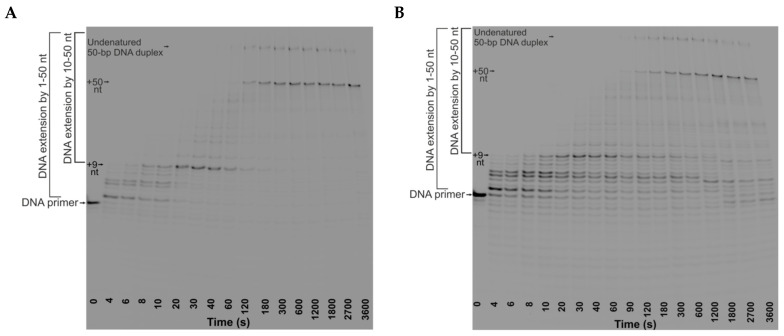
Representative PAGE analysis of DNA products. The time courses of DNA extension by Klenow fragment (1 μM) in Control substrate-11 (0.5 μM) in the presence of dNTPs (100 μM) (**A**) and in TRC-substrate-11 (0.5 μM) in the presence of dNTPs (100 μM) and RNAP (1 μM) (**B**).

**Figure 5:**
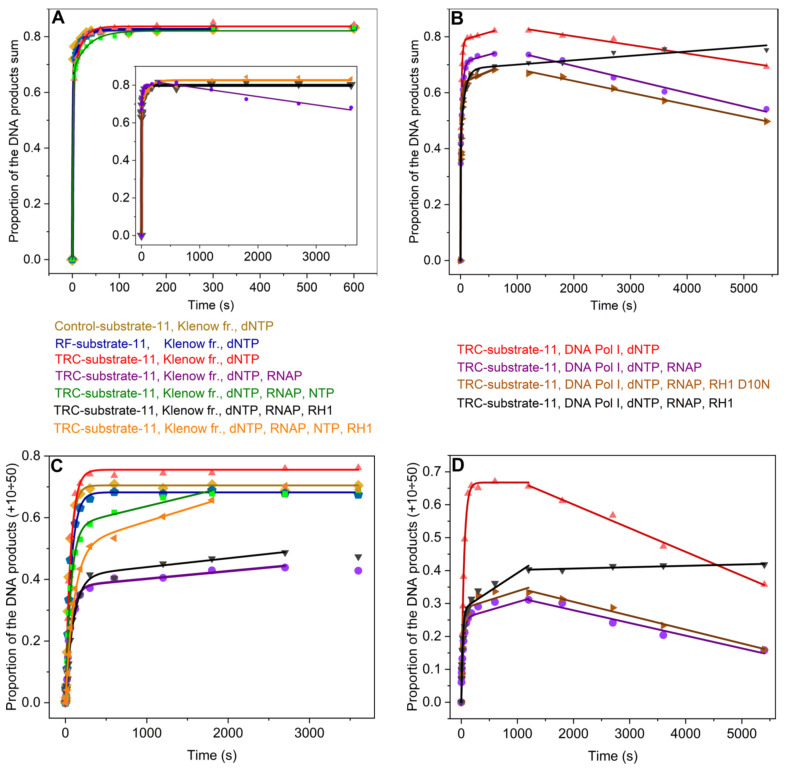
DNA extension by DNA polymerase (1 μM) in the absence or presence of RNAP (1 μM), RNase H1 (RH1) (3 μM), and RNase H1 D10N (RH1 D10N, the catalytically inactive mutant form of RH1 that lacks the catalytic activity but retains the ability to bind the substrate) (3 μM). The substrate concentration was 0.5 μM. DNA extension by 1–50 nt by Klenow fragment (**A**) and DNA Pol I (**B**). DNA extension by 10–50 nt by Klenow fragment (**C**) and DNA Pol I (**D**). The time courses of DNA extension by Klenow fragment in Control-substrate-11 (light brown), in RF-substrate-11 (blue), in TRC-substrate-11 (red), in TRC-substrate-11 in the presence of RNAP (purple), in TRC-substrate-11 in the presence of RNAP and NTPs (green), in TRC-substrate-11 in the presence of RNAP and RH1 (black), in TRC-substrate-11 in the presence of RNAP, NTPs, and RH1 (orange). The time courses of DNA extension by DNA Pol I in TRC-substrate-11 (red), in TRC-substrate-11 in the presence of RNAP (purple), in TRC-substrate-11 in the presence of RNAP and RH1 D10N (brown), in TRC-substrate-11 in the presence of RNAP and RH1 (black).

The efficiency and the rate of DNA extension by DNA polymerase in the TRC complexes were also determined from kinetic traces of the accumulation of DNA products by the Klenow fragment or DNA Pol I in the presence of RNAP (Figure 4 and Figure 5). The reaction mixtures either contained the NTPs or did not. In addition, to establish the basis for comparison of DNA polymerase action in the presence and absence of RNAP, we obtained time courses of DNA extension in the absence of RNAP. We used TRC-substrate-11, RF-substrate-11 (which contains the replication fork but lacks the RNA primer) as well as Control-substrate-11 (which consists of a nontemplate DNA strand and a DNA primer) (see Table 1).

The kinetic traces of accumulation of total products by Klenow fragment in the absence of RNAP or in the presence of RNAP elongating the RNA (in the presence of NTPs) showed the product fraction increase reaching a plateau. These kinetic traces were fitted to biexponential Equation (3) and yielded the observed rate constants $k_{1exp}^{DNA\;Sum}$, $k_{2exp}^{DNA\;Sum}$ and the formation efficiency of the enzyme–substrate complexes [ES]^DNA Sum^ (Figure 5A, Table 3). At the same time, the kinetic traces of accumulation of total products by Klenow fragment in the presence of stalled RNAP (without NTPs) showed an increase in the product fraction within the time range from 0 to 300 s with a kink at ~300 s, followed by a slow linear decrease in product fraction. Thus, the head-on collision of the Klenow fragment with RNAP stalled in the complex with the R-loop shifted the equilibrium accumulation of total DNA products towards the products of the 3′⟶5′ exonuclease activity of DNA polymerase at times exceeding 300 s. Within the time range from 0 to 300 s, the kinetic traces were also fitted to biexponential Equation (3). The further linear decrease in product fraction was fitted to linear Equation (5) and yielded the observed rate constant $k_{exo}^{DNA\;Sum}$. This constant characterizes the 3′⟶5′ exonuclease activity of DNA polymerase, which leads to a decrease in the total amount of DNA products (Table 3). We did not observe a similar decrease in total amount of DNA products during head-on collision between the Klenow fragment and an elongating RNAP. This is likely because the template and non-template DNA strands separate during the movement of RNAP and DNA polymerase toward each other.

The kinetic traces of accumulation of total products by full-length DNA Pol I in TRC-substrate-11 both in the presence and absence of RNAP showed an increase in the product fraction within the time range from 0 to 600 s with a kink at ~600 s, followed by a slow linear decrease in product fraction (Figure 5B). Consequently, in the case of DNA Pol I, the equilibrium accumulation of DNA products in TRC-substrate-11 was shifted towards the products of the exonuclease activity of DNA polymerase at times exceeding 600 s irrespective of whether RNAP was present or not. The kinetic traces were fitted to Equation (4) within the time range from 0 to 600 s: biexponential growth was followed by linear growth (Table 3). The latter slow linear growth corresponded to the steady-state accumulation of total DNA products and was characterized by the observed rate constant $k_{line}^{DNA\;Sum}$. The further linear decrease was fitted to linear Equation (5) and yielded the observed rate constant $k_{exo}^{DNA\;Sum}$ characterizing the 3′⟶5′ exonuclease activity of DNA polymerase.

Figure 4 clearly shows that the first ~5 nucleotides are incorporated much faster than the following ones. Moreover, this is true for TRC-substrate-11, RF-substrate-11, and Control-substrate-11 in the absence and presence of RNAP. Thus, the lower rate of accumulation of total DNA products compared to initial short products is not associated with the presence of the R-loop or RNAP. This is probably caused by DNA polymerase pausing depending on the sequence of the nontemplate DNA strand. Most of the pauses were observed prior to the incorporation of purine deoxynucleosides (Figure 4, Table 1). The rate constant $k_{1exp}^{DNA\;Sum}$ characterizes the pre-steady-state accumulation of initial short DNA products up to ~+5 nt while the rate constant $k_{2exp}^{DNA\;Sum}$ characterizes the pre-steady-state accumulation of total DNA products up to +50 nt. Total products accumulate more than an order of magnitude slower than the initial short ones.

The presence of RNAP (both in the absence and presence of NTPs) did not affect the accumulation of initial short DNA products by Klenow fragment or the formation efficiency of the complex of Klenow fragment with TRC-substrate-11, since the values of $k_{1exp}^{DNA\;Sum}$ and [ES]^DNA Sum^ were the same in the presence and absence of RNAP. At the same time, the observed rate constant of the accumulation of total DNA products $k_{2exp}^{DNA\;Sum}$ was found to decrease 1.3-fold in the presence of stalled RNAP (in the absence of NTPs) and 1.8-fold in the presence of RNAP together with NTPs. It can be suggested that the stalled RNAP stimulates Klenow fragment pausing in the pre-steady-state phase of head-on TRC. Addition of NTPs to the reaction mixture promotes the movement of RNAP along TRC-substrate-11 towards DNA polymerase, which enhances Klenow fragment pausing in the pre-steady-state phase.

In the case of full-length DNA Pol I, the presence of RNAP did not affect the formation efficiency of the complex of the enzyme and substrate, since the value of [ES]^DNA Sum^ was the same in the presence and absence of RNAP. On the other hand, the head-on collision of DNA Pol I with stalled RNAP probably stimulated DNA polymerase pausing during the pre-steady-state accumulation of DNA products, since the values of $k_{1exp}^{DNA\;Sum}$ and $k_{2exp}^{DNA\;Sum}$ decreased 1.4-fold and 1.6-fold, respectively, upon addition of RNAP to the reaction mixture. The head-on collision of DNA Pol I (but not the Klenow fragment) with stalled RNAP influenced the pre-steady-state accumulation of initial short DNA products up to ~+5 nt, probably due to its 5′⟶3′ exonuclease domain, since this domain causes DNA Pol I to encounter RNAP faster than the Klenow fragment. At the same time, addition of RNAP did not influence the subsequent steady-state extension of total DNA products by DNA Pol I in TRC-substrate-11 (see $k_{line}^{DNA\;Sum}$ in Table 3). Moreover, our data suggested that stalled RNAP enhanced the exonuclease activity of DNA Pol I in the complex with TRC-substrate-11, since the observed rate constant of the accumulation of exonuclease products ($k_{exo}^{DNA\;Sum}$) was found to increase 1.5-fold when RNAP was added to the reaction mixture.

The replication fork within our TRC substrate is located at a distance of 20 nt from the 3′ end of RNA primer (Table 1). It is known that the DNA-binding site of RNAP interacts with 2 unpaired nt and ~9 bp of downstream DNA [14]. Thus, in our model system, +10-th deoxynucleoside can attach to nascent DNA only if DNA polymerase does not collide with RNAP bound in the competent TEC. Therefore, we also analyzed the accumulation of DNA products from +10 to +50 nt to monitor the collision of DNA polymerase with RNAP (Figure 5C,D).

It turned out that the time courses of the accumulation of DNA products from +10 to +50 nt by Klenow fragment were monophasic in the absence of RNAP (Figure 5C). Thus, these time courses were fitted to single-exponential Equation (1) and yielded the proportion of the initial catalytically competent enzyme–substrate complex, where the DNA primer was extended by 10–50 nt ([ES]^DNA [10÷50]^), and the observed rate constant characterizing the pre-steady-state accumulation of DNA products from +10 to +50 nt ($k_{1exp}^{DNA\;[10÷50]}$) (Table 4). In the case of DNA Pol I, the appropriate time course was also monophasic within the time range from 0 to 1200 s (Figure 5D). The time course then showed a kink, followed by a slow linear decrease in the product proportion. Thus, the increase in the product proportion in the latter kinetic trace was fitted to single-exponential Equation (1). The further linear decrease was fitted to linear Equation (5) and yielded the observed rate constant characterizing the 3′⟶5′ exonuclease degradation of DNA products from +10 to +50 nt by DNA polymerase ($k_{exo}^{DNA\;\left[10÷50\right]}$) (Table 4). The time courses of the accumulation of DNA products by Klenow fragment from +10 to +50 nt became diphasic when RNAP was added and had burst-like traces. Therefore, the kinetic traces were fitted to Equation (2) (single-exponential growth followed by linear growth) within the time range from 0 to 2700 s (Figure 5C) and yielded the observed rate constants $k_{1exp}^{DNA\;[10÷50]}$ (characterizing the pre-steady-state accumulation of DNA products from +10 to +50 nt) and $k_{1line}^{DNA\;[10÷50]}$ (characterizing the steady-state accumulation of DNA products from +10 to +50 nt) (Table 4). At the same time, in the case of DNA Pol I, in the presence of RNAP, the time course showed a diphasic increase in the product proportion within the time range from 0 to 1200 s with a further kink, followed by a slow linear decrease in product proportion (Figure 5D). Thus, the latter kinetic trace was fitted to Equation (2) (single-exponential growth followed by linear growth) within the time range from 0 to 1200 s. The further linear decrease was fitted to linear Equation (5) (Table 4). Consequently, in the case of DNA Pol I, the equilibrium accumulation of products from +10 to +50 nt was shifted towards the products of the 3′⟶5′ exonuclease activity of DNA polymerase over long times both in the presence and absence of RNAP.

On the other hand, in the case of the Klenow fragment, we did not observe a backward shift in its equilibrium accumulation of products from +10 to +50 nt (Figure 5C), whereas its equilibrium accumulation of total DNA products was shifted backwards in the presence of stalled RNAP (without NTPs) (Figure 5A). Taken in tandem, these data suggest that a stalled TEC shifts the equilibrium accumulation of DNA products towards the products of the 3′⟶5′ exonuclease activity of the Klenow fragment when the Klenow fragment replicates DNA downstream of the stalled TEC.

The proportion of the initial catalytically competent DNA polymerase complex with TRC-substrate-11, where the DNA primer was extended by 10–50 nt ([ES]^DNA [10÷50]^) decreased 2.1-fold in the case of the Klenow fragment and 2.6-fold in the case of DNA Pol I when RNAP was added (Table 4). During RNAP interaction with the TRC-substrate-11, the proportion of the initial catalytically competent TEC ([TEC]^Sum^) did not exceed 62% in the absence of DNA polymerase or in the presence of Klenow fragment and did not exceed 72% in the presence of DNA Pol I (Table 2). At the same time, in the presence of RNAP, the proportion of the initial catalytically competent DNA polymerase complex with TRC-substrate-11 ([ES]^DNA [10÷50]^), where the DNA primer was extended by more than 9 nt, was 37% in the case of the Klenow fragment and 25.5% in the case of DNA Pol I (Table 4). Hence, the DNA extension in the TRC-substrate-11 by more than 9 nt presumably occurred in the proportion of the substrate that was not bound in the catalytically competent TEC. The burst phase in the kinetic traces of accumulation of DNA products from +10 to +50 nt probably corresponded to the DNA extension within the complex of DNA polymerase with the substrate initially unbound in the catalytically competent TEC. Consequently, the head-on conflict of replication with transcription occurs in our model system: RNAP stalled in the catalytically competent TEC interferes with replication.

Consequently, our data reveal that while stalled RNAP poses a strong roadblock for DNA polymerase, stalled Pol I does not obstruct RNAP. RNAP can displace a stalled DNA polymerase during elongation, but DNA Pol I lacks the ability to displace a stalled RNAP. This can probably be explained by the fact that the stability of the catalytically competent RNAP complex with an R-loop is higher than the stability of the DNA polymerase complex with its substrate. This assumption is consistent with data showing that the processivity of RNAP (10^3^–10^5^ nucleotides [24]) is significantly higher than that of DNA Pol I (no more than 188 nucleotides [25]). 

On the other hand, the proportion of the initial catalytically competent complex of Klenow fragment with TRC-substrate-11 ([ES]^DNA [10÷50]^), where the DNA primer was extended by more than 9 nt, decreased only 1.3-fold in the presence of RNAP together with NTPs (Table 4). Moreover, in the latter case, the proportion of DNA products extended by more than 9 nt reached the level seen without RNAP within 1800 s (Figure 5C). It can be proposed that after RNAP displaces the DNA polymerase and reaches the end of the DNA template strand, the DNA polymerase can reassociate with the nontemplate DNA strand (in a complex with the DNA primer) upstream of RNAP and resume DNA elongation, thus overcoming the head-on conflict between replication and transcription. This overcoming was characterized by the observed rate constant of steady-state accumulation of DNA products from +10 to +50 nt $k_{1line}^{DNA\;[10÷50]}$, which was more than three orders of magnitude lower than the pre-steady-state accumulation of these DNA products (compare $k_{1exp}^{DNA\;[10÷50]}\;and\;k_{1line}^{DNA\;[10÷50]}$ in Table 4). In addition, in ~5% of TRC complexes, the head-on collision of the Klenow fragment or full-length DNA Pol I with stalled RNAP was overcome (Figure 5C,D, $k_{1line}^{DNA\;[10÷50]}$ in Table 4). In these ~5% of the TRC complexes, RNAP probably formed an incompetent complex with the R-loop, which did not stall replication but stimulated DNA polymerase pausing, resulting in a decrease by more than three orders of magnitude in the rate of accumulation of DNA products from +10 to +50 nt (compare $k_{1exp}^{DNA\;[10÷50]}\;and\;k_{1line}^{DNA\;[10÷50]}$ in Table 4). The collision between the Klenow fragment and stalled RNAP (occurring in ~5% of TRC complexes) was resolved at a rate two times lower than its collision with an actively elongating RNAP ($see\;k_{1line}^{DNA\;[10÷50]}$ in Table 4).

At the same time, the proportion of the catalytically competent complex of Klenow fragment [ES]^DNA [10÷50]^ with the R-loop-containing substrate (TRC-substrate-11) and the rate constant of DNA extension by 10–50 nt ($k_{1exp}^{DNA\;[10÷50]}$) in this substrate were the same as in the case of complexes with substrates lacking an R-loop (RF-substrate-11 and Control-substrate-11) (Table 4). Analysis of the accumulation of DNA products from +21 to +50 nt gave the same result (Data not shown). Hence, an R-loop containing an 11-nt bubble in the absence of RNAP did not interfere with replication by Klenow fragment. On the other hand, in the case of full-length DNA Pol I, an R-loop containing an 11-nt bubble in the absence of RNAP also did not stall the replication but shifted the equilibrium accumulation of total DNA products and products from +10 to +50 nt towards the products of the 3′⟶5′ exonuclease activity of DNA polymerase. There is a broad understanding of R-loops as a replication fork barrier. However, RNAP was present in the experimental systems used in all studies where the R-loop was shown to be a replication obstacle [20,26]. The results obtained in this study reveal that the isolated R-loop itself does not stall replication by DNA Pol I. The polymerase resolves the R-loop, probably due to its strand displacement activity. Thus, it is the presence of RNAP forming the catalytically competent TEC with the R-loop that interferes with replication by DNA Pol I.

Therefore, our data reveal that the head-on conflict of replication with transcription has been registered in our model system: RNAP stalled in the catalytically competent TEC with an R-loop containing an 11-nt bubble (R-loop-11) interferes with the action of DNA polymerases both lacking and possessing 5′⟶3′ exonuclease activity.

What is more, the stalled RNAP stimulates a pause of the Klenow fragment in the pre-steady-state phase of head-on TRC and the 3′⟶5′ exonuclease activity of the Klenow fragment in the steady-state phase when the Klenow fragment replicates DNA downstream of the stalled RNAP. It was shown that it is the incompetent complex of RNAP with a TRC substrate that stimulates the 3′⟶5′ exonuclease activity of the Klenow fragment during a head-on TRC. Furthermore, when RNAP moves along TRC-substrate-11 towards the Klenow fragment, elongating the RNA, the pausing of the Klenow fragment is enhanced in the pre-steady-state phase; then, in the steady-state phase, the head-on conflict of replication with transcription is slowly overcome in our model system, likely through the reassociation of the displaced DNA polymerase with the nontemplate DNA strand upstream of RNAP. At the same time, our data reveal that free R-loop-11 does not interfere with DNA replication by the Klenow fragment.

In the case of full-length DNA Pol I, the steady-state accumulation of DNA products in TRC-substrate-11 is shifted towards the products of the exonuclease activity of DNA polymerase at times exceeding 600 s irrespectively of whether RNAP was present or not. Thus, R-loop-11 in the absence of RNAP does not stall replication but shifts the equilibrium accumulation of DNA products towards the products of the 3′⟶5′ exonuclease activity of DNA polymerase. The head-on collision of DNA Pol I with stalled RNAP stimulates DNA polymerase pausing during pre-steady-state accumulation of DNA products and enhanced 3′⟶5′ exonuclease activity of DNA Pol I in the steady-state phase. It was shown that the incompetent complex of RNAP with TRC-substrate enhanced the 3′⟶5′ exonuclease activity of DNA Pol I.

Both DNA polymerases used in the study slowly overcame a head-on conflict with stalled RNAP in only ~5% of TRC complexes in our model system. In these ~5% of the TRC complexes, RNAP probably formed an incompetent complex with the R-loop, which does not stall replication but rather stimulates DNA polymerase pausing in the steady-state phase, resulting in a decrease by more than three orders of magnitude in the rate of accumulation of DNA products from +10 to +50 nt.

### 2.4. RNA Cleavage by RNase H1 (RH1) in the TRC Substrate

**Figure 6:**
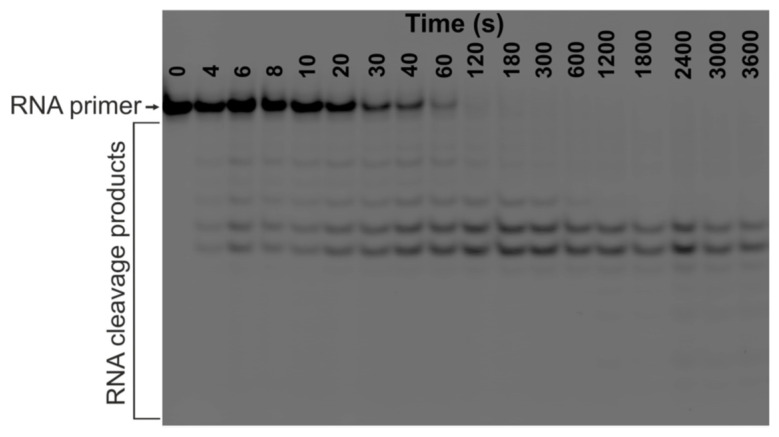
Representative PAGE analysis of RNA cleavage products. The time course of RNA cleavage by **RH1** (3 μM) in TRC-substrate-11 (0.5 μM) in the absence of RNAP, DNA Pol I, and dNTPs.

**Figure 7:**
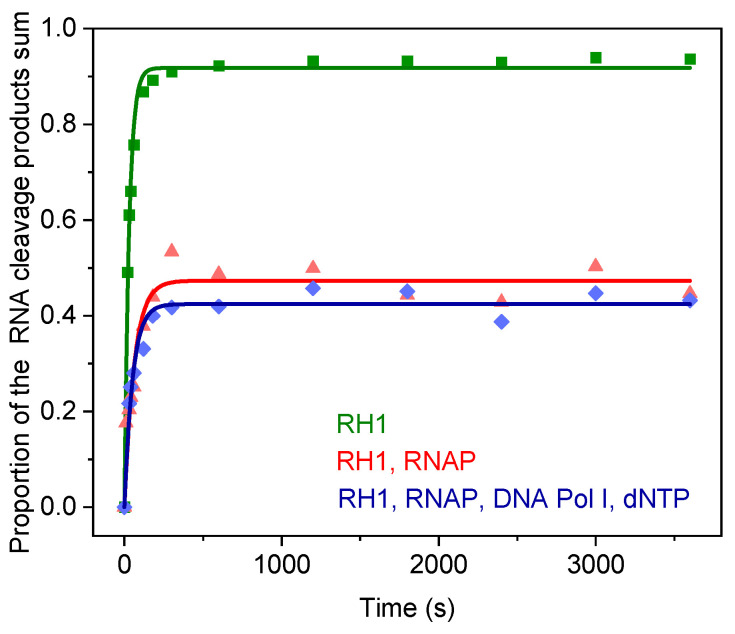
The time courses of RNA cleavage in TRC-substrate-11 (0.5 μM) by RH1 (3 μM) (green), by RH1 (3 μM) in the presence of RNAP (1 μM) (red), or RNAP, DNA Pol I (1 μM), and dNTPs (100 μM) (blue). Time courses were fitted to single-exponential Equation (1).

RNase H1 (RH1) cleaves the RNA strand within a hybrid RNA–DNA duplex. Thus, this enzyme can participate in R-loop degradation [27]. Kinetic traces of RNA cleavage by RH1 in TRC-substrate-11 in the presence of RNAP or RNAP, DNA Pol I, and dNTPs were determined. In addition, to establish the basis for comparison of RH1 action in the presence and absence of additional enzymes, we obtained kinetic traces of RNA cleavage in TRC-substrate-11 free of RNAP and DNA polymerase (Figure 6 and Figure 7).

We determined the dissociation constant of the RH1 D10N complex with the R-loop containing the 11-nt bubble of 1.2 ± 0.4 μM in our previous study [27]. RH1 D10N is the catalytically inactive mutant form of the enzyme that lacks catalytic activity but retains the ability to bind a substrate [28]. Therefore, in the present study, we increased the concentration of RNase H1 up to 3 μM in the reaction mixture. This increase in concentration allowed us to obtain at least 69% initial substrate binding when RNase H1 interacted with free TRC-substrate-11.

The kinetic traces of the accumulation of total products of RNA cleavage in the TRC-substrate-11 were monophasic and were therefore fitted to single-exponential Equation (1) (Figure 7, Table 5).

As can be seen from Table 5, the proportion of the catalytically competent RH1 complex with TRC-substrate-11 ([ES]^RNA Cleav^), where the RNA primer was cleaved, decreased two-fold when RNAP was added and 2.2-fold when RNAP, DNA Pol I, and dNTPs were added. Thus, the RNA cleavage in TRC-substrate-11 probably occurred in the proportion of the substrate that was not bound in the catalytically competent TEC. Thus, RH1 did not displace stalled RNAP from the competent complex with the R-loop containing an 11-nt bubble (enzyme–R-loop complex in the post-translocated state), even though the concentration of RH1 was high. Furthermore, RH1 also failed to displace stalled RNAP during head-on TRC, when a DNA polymerase, while extending the DNA strand, collided with a competent TEC. At the same time, the observed rate constant of RNA cleavage by RH1 in the TRC-substrate-11 ($k_{1}^{RNA\;Cleav}$) decreased 2.1-fold upon the addition of RNAP to the reaction mixture and 1.8-fold during head-on TRC. This ~2-fold rate decrease can probably be explained by RH1 displacing RNAP from its incompetent complex with the R-loop (probably an enzyme–R-loop complex in a backtracked state) before RNA cleavage.

### 2.5. DNA Extension by DNA Polymerase in the TRC Substrate in the Presence of RH1

To analyze the ability of RH1 to overcome a head-on conflict of replication and transcription, kinetic traces of accumulation of DNA products by DNA polymerase in the presence of RNAP and wild-type (wt) RH1 or its catalytically inactive mutant form RH1 D10N were obtained (Figure 5).

The kinetic traces of accumulation of total products by Klenow fragment or DNA Pol I in the presence of stalled RNAP (without NTPs) and wt RH1 did not show a kink followed by a slow linear decrease in product proportion, which was observed in the absence of RH1. The kinetic traces showed an increase in the product fraction. In the case of Klenow fragment, the kinetic trace was fitted to the biexponential Equation (3) and yielded the observed rate constants $k_{1exp}^{DNA\;Sum}$, $k_{2exp}^{DNA\;Sum}$ and the formation efficiency of enzyme–substrate complexes [ES]^DNA Sum^ (Figure 5A, Table 3). In the case of full-length DNA Pol I, the kinetic trace in the presence of RH1 was fitted to Equation (4): biexponential growth was followed by linear growth (Figure 5B, Table 3). The latter slow linear growth was characterized by the observed rate constant $k_{line}^{DNA\;Sum}$. Thus, the addition of wt RH1 to the reaction mixture eliminated the shift in the equilibrium accumulation of total DNA products towards the products of 3′⟶5′ exonuclease activity of DNA polymerase in the case of head-on TRC. On the other hand, RH1 D10N did not affect significantly the kinetic traces of accumulation of DNA products by DNA Pol I in TRC-substrate-11 in the presence of stalled RNAP (Figure 5B,D, Table 3 and Table 4). The appropriate kinetic traces in the presence of RH1 D10N were fitted to the same equations as in its absence. Consequently, the effect of wt RH1 is due to its catalytic activity, not to substrate binding. At the same time, wt RH1 did not affect significantly the kinetic trace of accumulation of DNA products by the Klenow fragment in TRC-substrate-11 in the presence of RNAP and NTPs, probably due to the separation of DNA strands (Figure 5A,C).

In addition, wt RH1 barely influenced the time course of the accumulation of DNA products from +10 to +50 nt by Klenow fragment in TRC-substrate-11 in the presence of RNAP (Figure 5C, Table 4). The kinetic trace in the presence of RH1 was fitted to the same equation as in its absence (Equation (2)—single-exponential growth followed by linear growth). Since the accumulation of DNA products from +10 to +50 nt most probably occurred in the proportion of the substrate that was not bound in the catalytically competent TEC, the latter data indicated that a free R-loop containing an 11-nt bubble (R-loop-11) did not interfere with DNA replication by the DNA polymerase lacking a 5′⟶3′ exonuclease domain. On the contrary, in the cases of DNA Pol I, the addition of wt RH1 to the reaction mixture eliminated the shift in the steady-state accumulation of DNA products from +10 to +50 nt towards the products of the 3′⟶5′ exonuclease activity of DNA polymerase (Figure 5D). Thus, this kinetic trace was fitted to Equation (2) (single-exponential growth followed by linear growth) within the time range from 0 to 1200 s. The further slow linear increase was fitted to linear Equation (5) and yielded the observed rate constant $k_{2line}^{DNA\;[10÷50]}$. These data indicated that free R-loop shifted the steady-state accumulation of DNA products towards the products of the 3′⟶5′ exonuclease activity of full-length DNA polymerase.

In the case of TRC-substrate-11, the addition of wt RH1 did not increase the values of [ES]^DNA [10÷50]^ in the presence of RNAP to the level of those observed in the absence of RNAP either for the Klenow fragment or for DNA Pol I (Table 4). Consequently, wt RH1 alone did not provide a resolution of the head-on conflict of replication and transcription.

Our data revealed that RH1 did not displace stalled RNAP from the competent TEC. On the other hand, RH1 eliminated the shift in the steady-state accumulation of total DNA products towards the products of the 3′⟶5′ exonuclease activity of DNA polymerase during head-on TRC. The R-loop-11 in the absence of RNAP did not interfere with replication by the Klenow fragment. Thus, the cleavage of the free R-loop cannot explain the effect of RH1 on the 3′⟶5′ exonuclease activity of the Klenow fragment. Therefore, the only possible explanation of this effect is that RH1 displaces RNAP from the incompetent complex with the TRC substrate. Consequently, it is the incompetent complex of RNAP with the TRC substrate that stimulates the 3′⟶5′ exonuclease activity of the Klenow fragment (lacking 5′⟶3′ exonuclease activity) during head-on TRC. At the same time, in the case of full-length DNA Pol I (possessing 5′⟶3′ exonuclease activity), both a free R-loop and an incompetent complex of RNAP with TRC substrate stimulate 3′⟶5′ exonuclease activity of DNA polymerase. In addition, the observed rate constant of the pre-steady-state accumulation of total DNA products $k_{2exp}^{DNA\;Sum}$ by the Klenow fragment was found to decrease 1.5-fold upon the addition of RH1 to the TRC containing stalled RNAP (Table 3). In TRC containing stalled RNAP and full-length DNA Pol I extending DNA, the observed rate constants of pre-steady-state DNA products accumulation $k_{1exp}^{DNA\;Sum}$ and $k_{2exp}^{DNA\;Sum}$ and steady-state DNA products accumulation $k_{line}^{DNA\;Sum}$ decreased 1.3-fold, 2.4-fold, and 4-fold, respectively, upon the addition of RH1. It is likely that RH1 promotes the movement of RNAP towards DNA polymerase in the incompetent complex of RNAP with TRC substrate prior to the dissociation of RNAP from this complex, which enhances DNA polymerase pausing in the pre-steady-state phase. The co-addition of RH1 and NTPs to the TRC containing stalled RNAP and the Klenow fragment extending DNA had a more pronounced effect on DNA polymerase pausing in the pre-steady-state phase. The values of $k_{1exp}^{DNA\;Sum}$ and $k_{2exp}^{DNA\;Sum}$ decreased 1.3-fold and 4.5-fold, respectively. Presumably, RH1 promotes the movement of RNAP towards DNA polymerase in the incompetent complex of RNAP with TRC-substrate, whereas NTPs promote such movement in the competent TEC.

Therefore, the results displayed in this work clearly reveal that RH1 alone does not provide a resolution of a head-on conflict of replication and transcription in a complex containing a TRC-substrate with an 11-nt heteroduplex. Our data showed that RH1 does not displace stalled RNAP from a competent TEC containing an R-loop-11. Moreover, RH1 also does not displace stalled RNAP during head-on TRC, when DNA polymerase extending DNA collides with competent TEC. On the other hand, RH1 eliminates the shift in the steady-state accumulation of total DNA products towards the products of 3′⟶5′ exonuclease activity of DNA polymerase during head-on TRC, since RH1 probably displaces RNAP from the incompetent complex with the TRC substrate. The effect of wt RH1 was shown to be due to its catalytic activity rather than substrate binding. Furthermore, RH1 probably promotes the movement of RNAP towards DNA polymerase in the incompetent complex of RNAP with the TRC substrate prior to the dissociation of RNAP from this complex, which enhances DNA polymerase pausing in the pre-steady-state phase. The co-addition of RH1 and NTPs to the TRC containing stalled RNAP and the Klenow fragment, extending DNA, has a more pronounced effect on DNA polymerase pausing in the pre-steady-state phase. Presumably, RH1 promotes the movement of RNAP towards DNA polymerase in the incompetent complex of RNAP with TRC substrate, whereas NTPs promote such movement in a competent TEC.

## 3. Materials and Methods

### 3.1. Oligonucleotides Synthesis

The synthesis of the oligodeoxyribonucleotides and oligoribonucleotides (Table 1) was carried out on an ASM-800DNA/RNA synthesizer (Biosset, Novosibirsk, Russia). The oligodeoxyribonucleotides were purchased from Biosset. FAM-labeled oligoribonucleotides were synthesized by means of standard commercial phosphoramidites and CPG solid supports from Glen Research (Sterling, VA, USA) in the Laboratory of RNA Chemistry (ICBFM SB RAS, Novosibirsk, Russia). The oligonucleotides were deprotected according to the manufacturer’s protocols and were purified by high-performance liquid chromatography or denaturing 20% polyacrylamide gel electrophoresis (PAGE). The homogeneity of all used oligonucleotides was checked by PAGE. The concentrations of oligonucleotides were calculated from their absorbance at 260 nm (A_260_).

### 3.2. Enzyme Purification

*Escherichia coli* core RNAP was expressed and purified from BL21(DE3) cells transformed by a pVS10-based plasmid encoding all core RNAP subunits, as described previously [23].

### 3.3. Time Courses of RNA or DNA Extension and Cleavage in TRC Complexes

TRC Complexes containing the transcription elongation complex (TEC) and the replication fork (1 μM) were assembled, as described in [23,29]. To anneal the DNA/RNA heteroduplex or the DNA/DNA duplex, the mixtures were incubated for 3 min at 70 °C and allowed to cool slowly to room temperature. An RNA primer (1 μM) annealed to the template DNA (1 μM) was incubated with core RNAP (2 μM) for 10 min at 25 °C in a buffer, 40 mM Tris-HCl (pH 7.9), and 40 mM KCl. Then, the nontemplate DNA or the duplex of nontemplate DNA with DNA primer (1 μM) were added and incubated for 20 min at 25 °C. If necessary, DNA Pol I or Klenow fragment (2 μM) were added and incubated for 10 min at 25 °C.

The RNAP reaction was initiated by the rapid mixing of TRC complex (in a buffer, 40 mM Tris-HCl (pH 7.9), and 40 mM KCl) with an equal volume of 200 μM NTPs or the mixture of 200 μM NTPs with 200 μM dNTPs (in a buffer, 20 mM MgCl_2_, 40 mM Tris-HCl (pH 7.9), and 40 mM KCl) at 25 °C. The RNAP endonuclease reaction was initiated by the rapid mixing of TRC-complex (in a buffer, 40 mM Tris-HCl (pH 7.9), and 40 mM KCl) with an equal volume of buffer, 20 mM MgCl_2_, 40 mM Tris-HCl (pH 7.9), and 40 mM KCl at 25 °C. A FAM-labeled RNA primer was used for visualization.

The DNA polymerase reaction was initiated by the rapid mixing of TRC complex or complex of template and nontemplate DNA with DNA primer or duplex of nontemplate DNA with DNA primer (in a buffer, 40 mM Tris-HCl (pH 7.9), and 40 mM KCl) with an equal volume of 200 μM dNTPs or the mixture of 200 μM dNTPs with 200 μM NTPs (in a buffer, 20 mM MgCl_2_, 40 mM Tris-HCl (pH 7.9), and 40 mM KCl) at 25 °C. If necessary, the DNA polymerase reaction was initiated by the rapid mixing of the TRC complex (in a buffer, 40 mM Tris-HCl (pH 7.9), and 40 mM KCl) with an equal volume of the mixture of 200 μM dNTPs with 6 μM RNase H1 (or RNase H1 D10N) or the mixture of 200 μM dNTPs with 200 μM NTPs and 6 μM RNase H1 (in a buffer, 20 mM MgCl_2_, 40 mM Tris-HCl (pH 7.9), and 40 mM KCl) at 25 °C. A FAM-labeled DNA primer was used for visualization.

The RNase H1 reaction was initiated by the rapid mixing of the TRC complex (in a buffer, 40 mM Tris-HCl (pH 7.9), and 40 mM KCl) with an equal volume of 6 μM RNase H1 or the mixture of 6 μM RNase H1 with 200 μM dNTPs (in a buffer, 20 mM MgCl_2_, 40 mM Tris-HCl (pH 7.9), and 40 mM KCl) at 25 °C. A FAM-labeled RNA primer was used for visualization.

The reactions were quenched at designated intervals by 0.5 M EDTA. EDTA was removed from the samples using Sephadex G-25 (GE Healthcare, Uppsala, Sweden) saturated with 8 M Urea. RNA products were separated in 20% denaturing PAGE, visualized using the Amersham Typhoon Biomolecular Imager (GE Healthcare Bio-Sciences Corp., Westborough, MA, USA), and quantified using Gel-Pro Analyzer 4.0 software (Media Cybernetics, Rockville, MD, USA). Two or three individual traces were averaged for each reported curve.

The final concentrations of reactants in the reaction mixtures were as follows: 0.5 μM RNA primer, 0.5 μM template DNA, 0.5 μM nontemplate DNA, 1 μM core RNAP, 1 μM DNA Pol I (or Klenow fragment), 3 μM RNase H1 (or RNase H1 D10N), 100 μM NTPs, 100 μM dNTPs (or the mixture of 100 μM NTPs and 100 μM dNTPs) in a reaction buffer, 10 mM MgCl_2_, 40 mM Tris-HCl (pH 7.9), and 40 mM KCl.

### 3.4. PAGE Data Analysis

The time courses of the RNA or the DNA extension or cleavage obtained in the PAGE analysis were fitted to one of the following equations by means of OriginPro 2021 software (Originlab Corp., Northampton, MA, USA):

Product proportion = A × [1 − exp(−*k*_obs_ × t)] (1)

Product proportion = A × [1 − exp(−*k*_obs_^1^ × t)] + *k*_obs_^2^ × t (2)

Product proportion = B × [1 − exp(−*k*_obs_^1^ × t)] + D × [1 − exp(−*k*_obs_^2^ × t)] (3)

Product proportion = B × [1 − exp(−*k*_obs_^1^ × t)] + D × [1 − exp(−*k*_obs_^2^ × t)] + *k*_obs_^3^ × t (4)

Product proportion = C − *k*_obs_ × t (5)

where A is the amplitude of an initial burst phase corresponded to the amount of an initial catalytically competent enzyme–substrate complex, B and D are pre-exponential factors, *k*_obs_^i^ (s^−1^) denotes the observed rate constants, and t represents reaction time. The sum of B and D also corresponds to the amount of catalytically competent enzyme-substrate complex.

## 4. Conclusions

We developed a novel in vitro approach to recapitulate head-on transcription–replication conflicts (TRCs) with precise control over the reaction conditions. We analyzed the kinetics of RNA polymerase and DNA polymerase I during TRC, as well as the ability of RH1 to resolve this conflict.

In summary:

(1) It is a catalytically competent TEC that poses a strong roadblock to DNA polymerase progression.
(2) An incompetent (likely backtracked) RNAP complex with an R-loop, while not blocking fork progression, stimulates the 3′→5′ exonuclease activity and pausing of DNA polymerase.
(3) An isolated R-loop with an 11-nt heteroduplex does not stall replication, highlighting that the RNAP bound to it in the catalytically competent TEC is essential for DNA polymerase stalling.
(4) A stalled DNA polymerase does not obstruct transcription but stimulates the endonuclease activity of RNAP.
(5) RH1 does not resolve head-on transcription–replication conflict when DNA polymerase collides with a competent TEC. However, it displaces RNAP from an incompetent complex with an R-loop, presumably in cases where the RNAP has backtracked.

## Figures and Tables

**Table 1:** Sequences of oligonucleotide substrate used in this study *.

TRC-substrate-11:
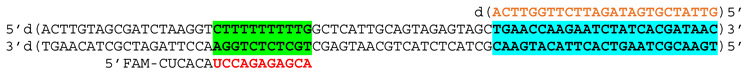
or
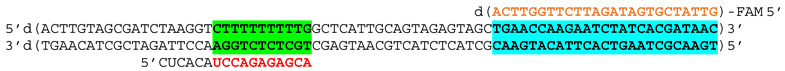
R-loop-substrate-11:
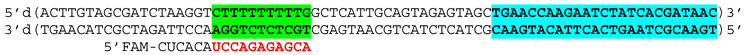
RF-substrate-11:
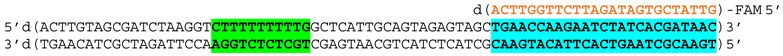
Control-substrate-11:
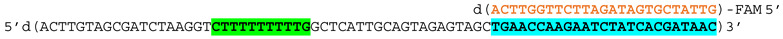

* The sequence forming a bubble is highlighted in green, the RNA sequence forming a DNA–RNA heteroduplex is indicated by red, the sequence forming a replication fork is highlighted in blue, and the DNA polymerase primer is indicated by orange.

**Table 2:** TEC formation efficiency and observed rate constants of RNA extension by RNAP.

Reaction Components	RNA Extension by 1–45 nt				RNA Extension by 21–45 nt			
	[TEC]^Sum^, %	$k_{1}^{RNA\;Sum}$, s^−1^	$k_{2}^{RNA\;Sum}$, s^−1^	$k_{endo}^{RNA\;Sum}$, s^−1^	[TEC]^21÷45^, %	$k_{1}^{RNA\;[21÷45]}$, s^−1^	$k_{2}^{RNA\;[21÷45]}$, s^−1^	$k_{endo}^{RNA\;[21÷45]}$, s^−1^
+RNAP +NTP +R-loop-substrate-11	52.7 ± 0.6	0.51 ± 0.06	(6.0 ± 0.9) × 10^−5^		18.3 ± 0.5	(32 ± 3) × 10^−3^	(1.6 ± 0.2) × 10^−5^	
+RNAP +NTP +TRC-substrate-11	55.7 ± 0.6	0.6 ± 0.1	(5.7 ± 0.9) × 10^−5^		17.9 ± 0.7	(21 ± 3) × 10^−3^	(2.4 ± 0.3) × 10^−5^	
+RNAP +NTP +TRC-substrate-11 +Klenow fragment	59.3 ± 0.4	0.59 ± 0.06	(4.3 ± 0.7) × 10^−5^		19.1 ± 0.7	(23 ± 3) × 10^−3^	(1.6 ± 0.3) × 10^−5^	
+RNAP +NTP +TRC-substrate-11 +Klenow fragment +dNTP	61.8 ± 0.9	0.50 ± 0.07	(8 ± 1) × 10^−5^		33.3 ± 0.8	Not determined	(8.5 ± 0.8) × 10^−3^	
+RNAP +NTP +TRC-substrate-11 +DNA Pol I	72.0 ± 0.9	0.7 ± 0.1	(13 ± 7) × 10^−5^	(2.1 ± 0.2) × 10^−5^	14.5 ± 0.6	(87 ± 8) × 10^−3^	(36 ± 6) × 10^−5^	(1.1 ± 0.2) × 10^−5^

**Table 3:** Formation efficiency of the complexes of DNA polymerase with substrates and observed rate constants of DNA extension by 1–50 nt.

Reaction Components	DNA Extension by 1–50 nt				
	[ES]^DNA Sum^, %	$k_{1exp}^{DNA\;Sum}$, s^−1^	$k_{2exp}^{DNA\;Sum}$, s^−1^	$k_{line}^{DNA\;Sum}$, s^−1^	$k_{exo}^{DNA\;Sum}$, s^−1^
+Klenow fragment, +dNTP +Control-substrate-11	82 ± 2	0.77 ± 0.07	0.06 ± 0.01		
+Klenow fragment, +dNTP +RF-substrate-11	83 ± 2	0.7 ± 0.1	0.058 ± 0.007		
+Klenow fragment, +dNTP +TRC-substrate-11	84 ± 4	0.8 ± 0.1	0.059 ± 0.008		
+Klenow fragment, +dNTP +TRC-substrate-11 +RNAP	80 ± 2	0.81 ± 0.07	0.045 ± 0.006		(4.4 ± 0.5) × 10^−5^
+Klenow fragment, +dNTP +TRC-substrate-11 +RNAP +NTP	82 ± 2	0.75 ± 0.08	0.032 ± 0.004		
+Klenow fragment, +dNTP +TRC-substrate-11 +RNAP +RNase H1	80 ± 4	0.8 ± 0.2	0.031 ± 0.007		
+Klenow fragment, +dNTP +TRC-substrate-11 +RNAP +NTP +RNase H1	83 ± 2	0.63 ± 0.08	0.01 ± 0.002		
+DNA Pol I, +dNTP +TRC-substrate-11	79 ± 1	0.69 ± 0.06	0.054 ± 0.001	(5.7 ± 0.5) × 10^−5^	(3.1 ± 0.2) × 10^−5^
+DNA Pol I, +dNTP +TRC-substrate-11 +RNAP	71 ± 2	0.51 ± 0.05	0.033 ± 0.002	(6 ± 1) × 10^−5^	(4.8 ± 0.4) × 10^−5^
+DNA Pol I, +dNTP +TRC-substrate-11 +RNAP +RNase H1	68 ± 3	0.38 ± 0.04	0.014 ± 0.002	(1.6 ± 0.3) × 10^−5^	
+DNA Pol I, +dNTP +TRC-substrate-11 +RNAP +RNase H1 D10N	63 ± 5	0.6 ± 0.3	0.041 ± 0.006	(8 ± 3) × 10^−5^	(3.8 ± 0.2) × 10^−5^

**Table 4:** Formation efficiency of the complexes of DNA polymerase with substrates and observed rate constants of DNA extension by 10–50 nt.

Reaction Mixture	[ES]^DNA [10÷50]^, %	$k_{1exp}^{DNA\;[10÷50]}$, s^1^	$k_{1line}^{DNA\;[10÷50]}$, s^−1^	$k_{2line}^{DNA\;[10÷50]}$	$k_{exo}^{DNA\;\left[10÷50\right]}$, s^−1^
+Klenow fragment, +dNTP +Control-substrate-11	70 ± 1	(1.8 ± 0.2) × 10^−2^			
+Klenow fragment, +dNTP +RF-substrate-11	68 ± 1	(1.5 ± 0.1) × 10^−2^			
+Klenow fragment, +dNTP +TRC-substrate-11	76 ± 2	(1.6 ± 0.2) × 10^−2^			
+Klenow fragment, +dNTP +TRC-substrate-11 +RNAP	37 ± 2	(1.5 ± 0.1) × 10^−2^	(3 ± 1) × 10^−5^		
+Klenow fragment, +dNTP +TRC-substrate-11 +RNAP +NTP	57 ± 2	(1.5 ± 0.1) × 10^−2^	(6 ± 2) × 10^−5^		
+Klenow fragment, +dNTP +TRC-substrate-11 +RNAP +NTP +RNase H1	51 ± 3	(1.0 ± 0.1) × 10^−2^	(8 ± 2) × 10^−5^		
+Klenow fragment, +dNTP +TRC-substrate-11 +RNAP +RNase H1	41 ± 2	(1.04 ± 0.09) × 10^−2^	(3 ± 1) × 10^−5^		
+DNA Pol I, +dNTP +TRC-substrate-11	66.7 ± 0.7	(2.09 ± 0.07) × 10^−2^			(7.2 ± 0.4) × 10^−5^
+DNA Pol I, +dNTP +TRC-substrate-11 +RNAP	25.5 ± 0.7	(2.7 ± 0.2) × 10^−2^	(5 ± 1) × 10^−5^		(3.9 ± 0.4) × 10^−5^
+DNA Pol I, +dNTP +TRC-substrate-11 +RNAP +RNase H1	32 ± 2	(4.0 ± 0.6) × 10^−2^	(11 ± 2) × 10^−5^	(4 ± 1) × 10^−6^	
+DNA Pol I, +dNTP +TRC-substrate-11 +RNAP +RNase H1 D10N	34 ± 3	(4.2 ± 0.5) × 10^−2^	(5 ± 2) × 10^−5^		(4.2 ± 0.2) × 10^−5^

**Table 5:** Formation efficiency of the complex of RNase H1 with TRC-substrate-11 and observed rate constants of RNA cleavage by RNase H1 in the TRC-substrate-11.

Reaction Mixture	RNA Cleavage (Products Sum)	
	[ES]^RNA Cleav^, %	$k_{1}^{RNA\;Cleav}$, s^−1^
+RNase H1 +TRC-substrate-11	91.8 ± 0.8	0.034 ± 0.003
+RNase H1 +TRC-substrate-11 +RNAP	47 ± 2	0.016 ± 0.003
+RNase H1 +TRC-substrate-11 +RNAP +DNA Pol I +dNTP	42 ± 1	0.019 ± 0.002

## Data Availability

The original contributions presented in this study are included in the article/Supplementary Materials. Further inquiries can be directed to the corresponding authors.

## Supplementary Materials

The following supporting information can be downloaded at: https://www.mdpi.com/article/10.3390/ijms262311515/s1.

## Abbreviations

The following abbreviations are used in this manuscript:

RNAP	RNA polymerase
DNA Pol I	DNA polymerase I
RH1	RNase H1
RH1 D10N	The catalytically inactive mutant form of RH1 that lacks the catalytic activity but retains the ability to bind the substrate
TRC	Transcription–replication conflict
TEC	Transcription elongation complex
R-loop-11	R-loop containing 11-nt heteroduplex
RF	replication fork
