# Supplementary material for: Interplay Between DNA Polymerase, RNA Polymerase, and RNase H1 During Head-On Transcription–Replication Conflict
Source: Int J Mol Sci. 2025 Nov 27;26(23):11515. doi: 10.3390/ijms262311515 (PMC12692348; doi:10.3390/ijms262311515)
Supplement: Supplementary file 1 [file ijms-26-11515-s001.zip › ijms-3992128-supplementary.pdf]

Interplay between DNA Polymerase, RNA Polymerase and RNase H1 during Head-on Transcription-Replication Conflict

Nadezhda A. Timofeyeva, Ekaterina I. Tsoi, Darya S. Novopashina, Nikita A. Kuznetsov and Aleksandra A. Kuznetsova

SUPPLEMENTARY INFORMATION

Formation of the Pre-Translocated Complex of RNAP with an R-Loop

To determine the equilibrium dissociation constant characterizing the stability of the catalytically competent TEC in the pre-translocated state ($\boldsymbol{K_d^*}$), we analyzed the kinetics of RNA extension in the R-loop containing the 9 nt bubble (R-loop-9) in the presence of NTPs and various concentrations of RNAP (Figure S1A).

5′d(ACTTGTAGCGATCTAAGGTCT**TTTTTTTTG**GCTCATTGCAGTAGAGTAGC)3′
3′d(TGAACATCGCTAGATTCCAGA**GTCTCTCGT**CGAGTAACGTCATCTCATCG)5′ **R-loop-9**
5′-FAM-CUCACAUC**CAGAGAGCA**-3′

(the sequences forming a bubble are highlighted in green, the RNA sequence forming a DNA–RNA heteroduplex is indicated by red)

The kinetics of RNA elongation has burst-like traces (Figure S1B). The amplitude of the burst phase corresponds to the proportion of the catalytically competent TEC. A slow accumulation of the RNA product is following the burst phase. The kinetic traces (Figure S1B) were fitted to Equation (S1). As can be seen from Figure S1B, the amplitude of the burst phase is dependent on the concentration of RNAP. The dependence of the catalytically competent TEC proportion on RNAP concentration was fitted to Equation (S3) to obtain the dissociation constant ($\boldsymbol{K_d^*}$) of 0.8 ± 0.2 μM, characterizing the stability of the catalytically competent TEC in the pre-translocated state (Figure S1C). The obtained dissociation constant precises our earlier estimate of $\boldsymbol{K_d^*}$ (0.6 μM) reported in [1]. More precise determination of $\boldsymbol{K_d^*}$ confirms our earlier conclusion that the concentration of the catalytically competent TEC is lower than that of the overall RNAP complex with the R-loop at the initial moment as $\boldsymbol{K_d^*}$ value is 2.7-fold higher than the dissociation constant of the overall RNAP complex with this R-loop (0.3 μM) reported in [1].

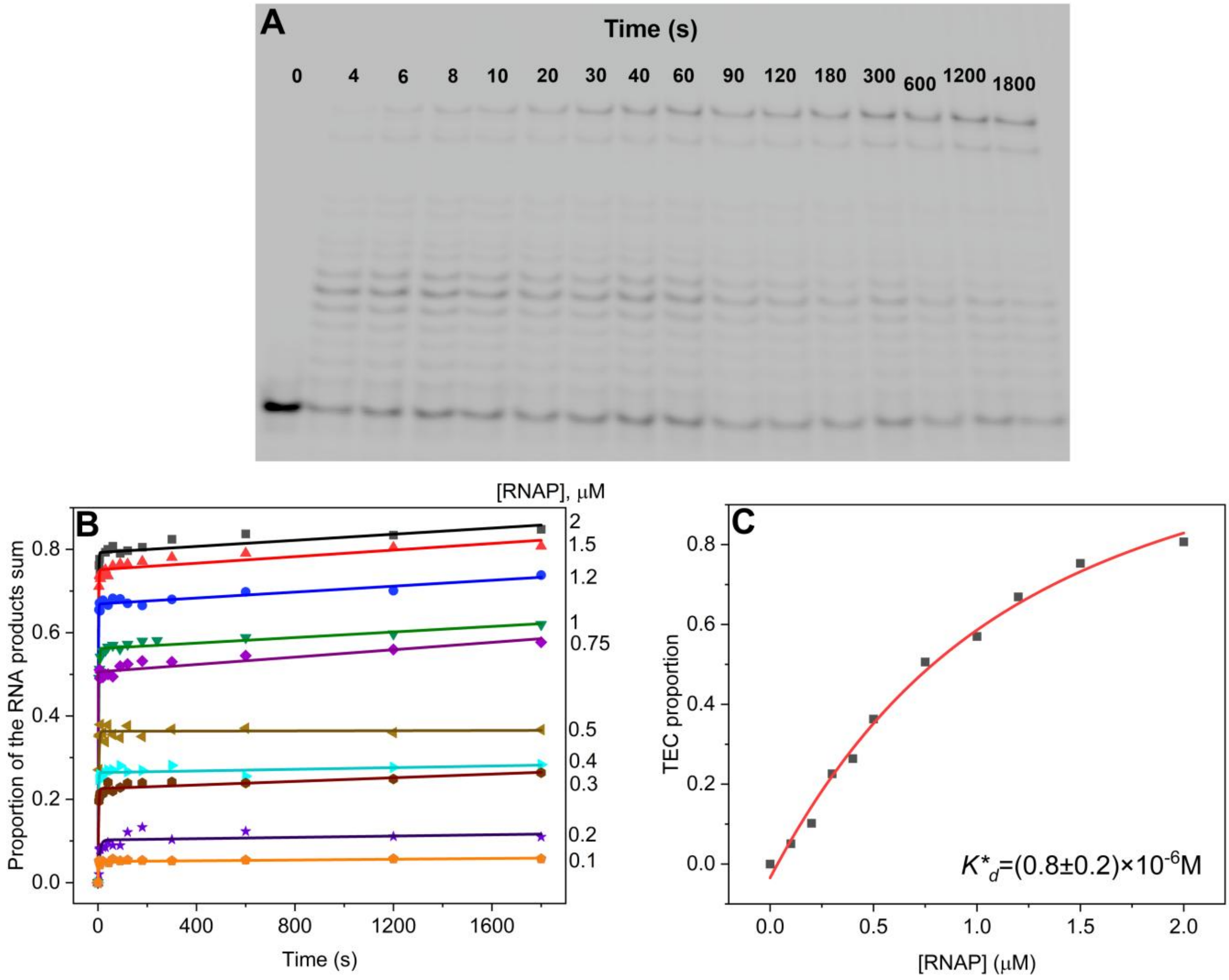

Figure S1. The determination of the dissociation constant characterizing the stability of the catalytically competent TEC in the pre-translocated state (K_d^*). (**A**) Polyacrylamide gel electrophoresis (PAGE) analysis of RNA extension in R-loop-9 (0.5 μM) by RNAP (1.2 μM). Time intervals for the enzymatic reaction are indicated above the corresponding lanes. (**B**) The time courses of RNA extension by 1–20 nt in R-loop-9 (0.5 μM). Concentrations of RNAP are indicated next to the right axis. Smooth curves are the result of the fitting procedure according to Equation (S1). The amplitude of an initial burst phase corresponds to the proportion of an initial catalytically competent TEC. (**C**) Dependence of the catalytically competent TEC proportion on the RNAP concentration in the reaction mixture. Data were fitted to Equation (S3) and yielded an equilibrium dissociation constant (K_d^*) of 0.8 ± 0.2 μM. Smooth curve is a result of the fitting procedure.

Materials and Methods

Time Courses of RNA Extension in TEC-Complex

To determine the dissociation constant of the catalytically competent transcription elongation complex between the RNAP and R-loop containing the 9 nt bubble, time courses of RNA extension in corresponding TEC-complex were obtained at various RNAP concentrations.

Transcription elongation complex was assembled, as described in [**Error! Bookmark not defined.**]. The RNAP polymerase reaction was initiated, quenched and analyzed, as described previously (see section 3.3 and [**Error! Bookmark not defined.**]). The final composition of reaction mixtures was as follows: 0.5 μM RNA primer, 0.55 μM template DNA, 1 μM nontemplate DNA, core RNAP at the required concentration, 100

μM NTPs in a reaction buffer, 10 mM $MgCl_2$, 40 mM Tris-HCl (pH 7.9), and 40 mM KCl. The concentration of RNAP was varied in the range 0.1-2.0 μM.

PAGE Data Analysis

The time courses of the RNA extension obtained in the PAGE analysis were fitted to the following equation by means of OriginPro 2021 software (Originlab Corp., Northampton, MA, USA):

$$\textit{Product proportion} = A \times [1 - \exp(-k_{obs}^{1} \times t)] + k_{obs}^{2} \times t \quad \text{(S1)}$$

where A is the amplitude of an initial burst phase corresponded to the amount of an initial catalytically competent enzyme-substrate complex, k_{obs}^{i} (s^{-1}) denotes the observed rate constants, and t represents reaction time.

The dependence of initial catalytically competent TEC proportion on the concentration of RNAP was used to calculate the dissociation constant K_d* according to the equations:

$$K_d^* = \frac{[E][S]}{[ES]^*} \quad \text{(S2)}$$

$$\frac{[ES]^*}{[S]_0} = \frac{([S]_0+[E]_0+K_d^*)-\sqrt{([S]_0+[E]_0+K_d^*)^2-4[E]_0[S]_0}}{2[S]_0} \quad \text{(S3)}$$

where $[S]_0$ and $[E]_0$ are total amounts of the R-loop and RNAP, respectively; [S], [E], and [ES]* are the concentrations of the free R-loop, enzyme, and their catalytically competent complex, respectively.

REFERENCES

1. Timofeyeva, N.A.; Tsoi, E.I.; Novopashina, D.S.; Kuznetsova, A.A.; Kuznetsov, N.A. Role of R-Loop Structure in Efficacy of RNA Elongation Synthesis by RNA Polymerase from *Escherichia coli*. *Int. J. Mol. Sci.* **2024**, Volume 25, p. 12190. https://doi.org/10.3390/ijms252212190.
